# A clinical pharmacogenetic characterization of DPD polymorphisms for pre-treatment screening of patients candidates to fluoropyrimidine therapy

**DOI:** 10.1186/1878-5085-5-S1-A24

**Published:** 2014-02-11

**Authors:** Marzia Del Re, Fotios Loupakis, Cecilia Barbara, Laura Lombardo, Tiziana Latiano, Elena Zafarana, Stefano Cordio, Luisa Toffolatti, Miriam Ricasoli, Dario Giuffrida, Jennifer Vanoli, Samantha Di Donato, Federico Grifalchi, Antonio Rinaldi, Alfredo Butera, Evaristo Maiello, Salvatore Siena, Alba Brandes, Alfredo Falcone, Federico Cappuzzo, Romano Danesi

**Affiliations:** 1 Department of Clinical and Experimental Medicine, University of Pisa, Italy; 2 Medical Oncology Unit 2, Azienda Ospedaliero Universitaria Pisana, Italy; 3 Medical Oncology Unit, Azienda USL6, Livorno, Italy; 4 Medical Oncology Unit, Bellaria Hospital, Bologna, Italy; 5 Medical Oncology IRCCS Casa Sollievo della Sofferenza, San Giovanni Rotondo, Italy; 6 Medical Oncology Unit, Ospedale Misericordia e Dolce, Prato, Italy; 7 Medical Oncology Unit, Garibaldi Hospital, Catania, Italy; 8 Medical Oncology Unit, Azienda Ospedaliera Vimercate, Monza, Italy; 9 Medical Oncology Unit, Azienda USL 3, Pistoia, Italy; 10 Medical Oncology Unit, Istituto Oncologico del Mediterraneo, Catania, Italy; 11 Medical Oncology Unit, Ospedale Niguarda Cà Granda, Milano, Italy; 12 Medical Oncology Unit, Ospedale MG Vannini, Roma, Italy; 13 Medical Oncology Unit, Azienda USL Ta1, Castellaneta, Italy; 14 Medical Oncology Unit, ASP Agrigento, Italy

## Background

DPD deficiency is the result of loss-of-function mutations within the dihydropyrimidine dehydrogenase (DPD) gene. The IVS14+1G>A variant is associated with DPD deficiency as a result of a 165-bp deletion in the DPD mRNA. A rare mutation, c.2846A>T, is characterized by a change of the acidic aspartic acid to the aliphatic valine with potential impairment of enzyme activity [1].

## Scientific objectives

In this study, we describe the spectrum of toxicities of fluoropyrimidines in patients carrying the IVS14+1G>A and 2846A>T variants.

## Technological approaches

Data were collected from 550 patients with gastrointestinal, breast, head-neck and pancreatic cancers. They were evaluated for DPD genotype upon development of grade ≥2 non-hematological and ≥3 hematological toxicities (CTCAE v.4) following standard fluoropyrimidine-containing regimens in combination with other cytotoxic agents and/or anti-EGFR and VEGF antibodies. DNA was extracted from blood by the Qiamp DNA Blood Mini Kit (Qiagen^®^) and IVS14+1G>A and 2846T>C DPD variants were screened on a Real-Time Life Sciences^®^ 7900 HT platform. The study was approved by the local Ethics Committee.

## Results interpretation

A total of 27 IVS14+1GA, five 2846AT, one IVS14+1AA and one 2846TT subjects were identified. Toxicities in all subjects were G3/4 diarrhea (100%), G3/4 mucositis (48%), febrile neutropenia (45%), G3/4 thrombocytopenia (38%), G3/4 anemia (24%), G2/3 hand-foot syndrome (14%), G3 dermatitis (7%) and G2/4 alopecia (7%). The IVS14+1AA patient showed diarrhea G2, mucositis G3, anemia G1, pistrinopenia G3, febrile neutropenia G4, complete alopecia and Staphylococcus aureus sepsis. This patient required 20 days of hospitalization and was managed with antibiotics, platelet transfusion, port removal, G-CSF administration and parenteral nutrition. The patient survived because she was given a reduced 5-FU 250 mg/sqm test dose without folates, while the 2846TT patient deceased after the first cycle of FOLFOX4 treatment because of a diarrhea G3, mucositis G4, febrile neutropenia and piastrinopenia G4.

## Outlook and expert recommendations

Patients carrying the deleterious IVS14+1G>A and 2846T>C variant alleles display severe toxicities which are fatal in homozygous variant subjects. Although the frequence of DPYD*2A allele is low, the screening for DPD mutation is clinically relevant to avoid the severe toxicities or death in patients treated with fluoropyrimidine-containing regimens. This finding suggests the usefulness of pre-treatment screening of DPD in patients candidates to fluoropyrimidine treatment.
